# A chemical biology/modular antibody platform for ADP-ribosylation signaling

**DOI:** 10.1016/j.tibs.2023.06.005

**Published:** 2023-10

**Authors:** Helen Dauben, Edoardo José Longarini, Ivan Matic

**Affiliations:** 1 Research Group of Proteomics and ADP-ribosylation Signaling, Max Planck Institute for Biology of Ageing, 50931 Cologne, Germany; 2 Cologne Excellence Cluster for Stress Responses in Ageing-Associated Diseases (CECAD), University of Cologne, 50931 Cologne, Germany

**Figure:**
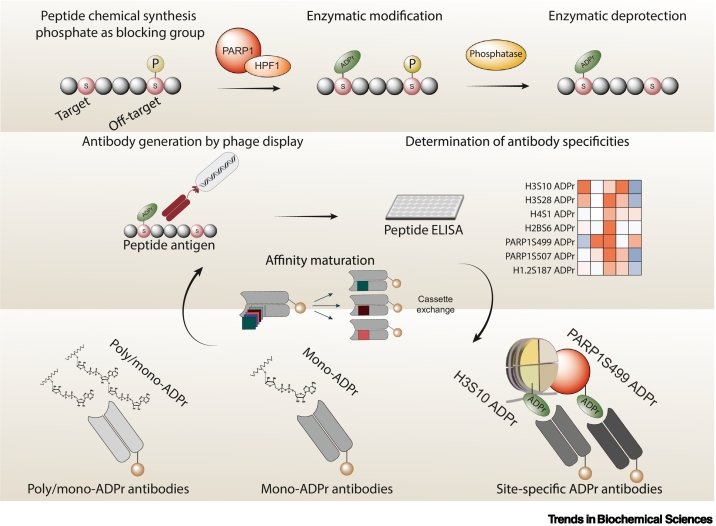

ADP-ribosylation (ADPr), a widespread post-translational modification, has crucial roles in various biological processes, particularly DNA repair signaling. Its clinical significance is underscored by the successful use of poly (ADP-ribose) polymerase (PARP) inhibitors in cancer therapy. Our recent discovery of serine ADPr by the HPF1/PARP1 complex has paved the way for a phospho-guided enzymatic strategy. This approach enables the site-specific installation of ADP-ribose on peptides that then serve as antigens for generating site-specific and broad-specificity mono-ADPr antibodies.

**Figure:**
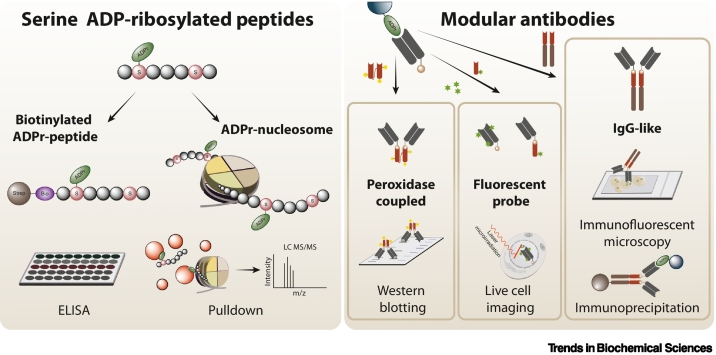

The sensitivity, versatility, and specificity of this strategy recently enabled our discovery that mono-ADPr is a second wave of PARP1 signaling, an unexpected finding given that, for over 50 years, poly-ADPr was viewed as the sole product of PARP1. Likewise, this methodology is poised to expedite our understanding of the signaling pathways regulated by various PARPs and other enzymes, most of which catalyze mono-ADPr.

## ADVANTAGES:

Fast production: numerous serine ADP-ribosylated peptides can quickly be generated in scalable amounts.

Broad applicability of serine ADP-ribosylated peptides: generation of diverse antibodies, peptide-based interaction proteomics, and preparation of site-specifically modified nucleosomes.

Remarkable mono-ADPr specificity: no cross-reactivity toward poly-ADPr.

Unique capability for antibody site-specificity: it is currently the only available strategy for generating site-specific ADPr antibodies.

Versatility: effortless format-switching for diverse applications, including a peroxidase-coupled format for superior sensitivity in western blotting.

Broad applicability of the antibodies beyond serine ADPr: recognition of mono-ADPr by other mono(ADP-ribose) transferases, including SIRT6, PARP3, and PARP14.

## CHALLENGES:

Limitations to the generation of site-specific antibodies targeting other residues: because the strategy is limited to the generation of serine and tyrosine ADP-ribosylated peptides, it falls short of creating site-specific antibodies for other forms of ADPr, such as on arginine, glutamate, and cysteine.

Limitations to poly-ADPr specificity: while our platform, currently based on mono-ADP-ribosylated peptides, can generate antibodies recognizing both mono- and poly-ADPr, creating antibodies that exclusively target poly-ADPr is not currently achievable.
